# Prevalence of Suicide-Related Consultations in Relation to the Total Number of Emergency Visits Attended by the Psychiatry Service at the University Hospital of Salamanca in 2023

**DOI:** 10.1192/j.eurpsy.2025.2347

**Published:** 2025-08-26

**Authors:** P. Andres-Olivera, E. Dominguez-Alvarez, C. P. Rodriguez, C. Munaiz-Cossio, R. K. Gonzalez-Bolaños, R. Brito-Rey, C. Marín-Lorenzo, B. Arribas-Simon

**Affiliations:** 1 Psichiatry, CAUSA; 2 Medicine, Usal; 3 CAUSA, Salamanca; 4 Psichiatry, HCUV, Valladolid, Spain

## Abstract

**Introduction:**

Suicide is a significant public health issue, and its study has been approached from various perspectives. This work focuses on analyzing consultations related to suicide attended by the Psychiatry Service at the University Hospital of Salamanca during 2023. Suicide is defined as an intentional act with a fatal outcome, and its prevention is considered a priority in mental health.

**Objectives:**

**General Objective:** To assess the prevalence of suicide-related consultations within the total psychiatric emergencies attended in 2023.

**Specific Objectives:**

1. To identify demographic and temporal patterns in suicide-related consultations.
2. To compare the prevalence of suicide-related consultations with other types of psychiatric emergencies attended during the same period.

**Methods:**

The study is observational and retrospective, based on the analysis of psychiatric emergency records from 2023. Demographic variables such as gender and age were analyzed, as well as the cause of the consultations, classified as “suicide-related” or “other psychiatric causes.” Statistical tools were used to identify significant patterns and relationships.

**Results:**

1. **Gender distribution:** 55.2% of the patients were women, and 44.8% were men.
2. **Age distribution:** Most consultations involved adults (74.4%), followed by older adults (16.7%) and minors (8.9%).
3. **Cause of the consultation:** 36.15% of the cases were related to suicidal behavior, while 63.85% were due to other psychiatric emergencies.
4. **Temporal pattern:** There was an increase in consultations during the autumn months, particularly in September and November.

**Conclusions:**

The study reveals that over one-third of psychiatric emergency consultations were related to suicide, underscoring the need to enhance prevention strategies and early intervention. The results also show significant differences by gender and age, as well as seasonal patterns that may be linked to emotional and social factors.

The findings emphasize the importance of identifying specific risk factors associated with gender and age, as well as reinforcing the training of emergency personnel to effectively intervene in cases of suicidal behavior. Moreover, attention should be given to months with higher incidence of suicide-related consultations, such as the autumn season.

**Disclosure of Interest:**

None Declared

